# “Having diabetes shouldn’t stop them”: healthcare professionals’ perceptions of physical activity in children with Type 1 diabetes

**DOI:** 10.1186/s12887-015-0389-5

**Published:** 2015-06-18

**Authors:** Helen Quirk, Holly Blake, Beatrice Dee, Cris Glazebrook

**Affiliations:** University of Nottingham, School of Health Sciences, Institute of Mental Health, Jubilee Campus, Triumph Road, Nottingham, NG7 2TU UK; University of Nottingham, School of Health Sciences, A Floor, South Block Link, Queen’s Medical Centre, Nottingham, NG7 2HA UK; University of Nottingham, Medical School, Queen’s Medical Centre, Nottingham, NG7 2UH UK; University of Nottingham, Division of Psychiatry and Applied Psychology, Institute of Mental Health, Jubilee Campus, Triumph Road, Nottingham, NG7 2TU UK

**Keywords:** Type 1 diabetes mellitus, Qualitative research, Child, Motor activity, Health personnel

## Abstract

**Background:**

Healthcare professionals (HCP) working with children who have Type 1 Diabetes Mellitus (T1DM) have an important role in advising about and supporting the control of blood glucose level in relation to physical activity. Regular physical activity has known benefits for children with T1DM, but children with chronic conditions may face barriers to participation. The perceptions of HCPs were explored in an effort to understand what influences physical activity in children with T1DM and to inform the practice of those working with children who have T1DM.

**Methods:**

Semi-structured interviews with 11 HCPs involved in the care of children with T1DM in the UK were conducted. Interviews were recorded, transcribed verbatim and data were analysed using thematic analysis.

**Results:**

The factors perceived to influence participation in physical activity are presented as five major themes and eleven sub-themes. Themes included the positive influence of social support, the child’s motivation to be active, the potential for formal organisations such as school and diabetes clinic to support physical activity, the challenges faced by those who have T1DM and the perceived barriers to HCPs fulfilling their role of promoting physical activity.

**Conclusions:**

Healthcare professionals recognised their role in helping children with T1DM and their parents to incorporate physical activity into diabetes management and everyday life, but perceived barriers to the successful fulfilment of this role. The findings highlight the potential for clinical and non-clinical supportive systems to be sensitive to these challenges and facilitate children’s regular participation in physical activity.

## Background

Regular physical activity for children and young people^1^ with Type 1 Diabetes Mellitus (T1DM) has been associated with improvements in glycaemic control, lipid profile and body composition [1]. Despite its potential to delay the onset of cardiovascular disease [2, 3], figures suggest that, in common with the general child population, children with T1DM are not meeting the recommended 60 minutes of moderate-to-vigorous physical activity per day [4–6]. Healthcare professionals (HCPs) potentially have an important role in encouraging children with T1DM to engage in regular physical activity. However, little is known about how HCPs view this role and factors influencing physical activity in this population.

Fear of hypoglycaemia has been identified as a barrier to physical activity in adults with T1DM [7], children with T1DM [8] and their parents [8–10]. Provision of support and guidance from HCPs is important for managing the risk of hypoglycaemia and alleviating associated worries [8], warranting the need to explore HCPs’ perceptions of the factors influencing physical activity in this population.

Theoretical models provide a framework for understanding these influential factors [11]. Bandura’s Social Cognitive Theory (SCT) suggests that the environment and the individual affect one another in a process of reciprocal determinism to bring about any given behaviour [12]. At the heart of SCT, self-efficacy refers to the belief in one’s ability to accomplish specific behaviours [12]. Although HCPs may not be able to alter the physical environment, they can be sensitive to the social influences involved in helping or hindering children’s participation in regular physical activity.

Furthermore parents of children with T1DM identify diabetes HCPs as an important influence on children’s physical activity. MacMillan and colleagues [6, 13] explored the perceptions of diabetes professionals together with teachers, youth with T1DM and their parents in Scotland to inform guidelines on how to improve physical activity support for young people with T1DM through diabetes care and the school setting. However, previous research has not distinguished HCPs’ beliefs from the views of other stakeholders, nor has there been an in-depth exploration of HCPs’ perceptions of the factors influencing the physical activity levels of children who have T1DM. Therefore, the purpose of this study was to understand HCPs’ perceptions of their role in relation to supporting physical activity for children with T1DM and their understanding of factors influencing levels of physical activity for children with T1DM in an effort to inform those working with children who have T1DM.

## Methods

Data were collected by semi-structured interview with HCPs involved in the care of children with T1DM. This study was informed by interrelated concepts of interpretivism and reflexivity, balanced with pragmatism and transparency. This was achieved by seeking to understand the perceptions of HCPs whilst demonstrating practical implications for those working with children who have T1DM. The research was reviewed and approved by the University of Nottingham Medical School Ethics Committee in January 2013 (Reference No: B10012013 SNMP).

Participants were recruited between February 2013 and March 2014 using a purposeful sampling approach with snowball techniques [14], whereby participants suggested colleagues who might be eligible. A recruitment poster was distributed amongst paediatric diabetes clinical network members and delegates at a professional diabetes conference. The poster contained a web link to a survey site [15] where potential participants could read about the study and provide their contact details confidentially. Those who provided their contact details were contacted by the lead researcher and eligible HCPs were provided with the study information sheet and a consent form.

The number of HCPs recruited to this study was based on the number needed to achieve theoretical data saturation [16]. With each interview, the researcher judged whether any new data were emerging that would satisfy the purpose of the research. Nineteen HCPs expressed an interest in being interviewed and no new data emerged during the tenth and eleventh interviews, at which point recruitment ceased.

The eleven participants were recruited from eight different paediatric diabetes centres across the UK, four participants were recruited from the same centre, but held different roles (Dietician, Clinical Lead Dietician, Consultant and Specialist Nurse). The sample consisted of four Consultants, six Dieticians and one Specialist Nurse with an average 7.2 years of experience working in paediatric T1DM (mean = 7.2 years, range = 1 – 17 years) among them. Interview length ranged from 18 to 85 minutes and the mean duration was 40 minutes.

Interviews were arranged for a mutually convenient time and location; telephone interviews were offered if a face-to-face interview was not feasible. Two interviews were conducted face-to-face at the local institution, nine were conducted by telephone. The interviewer received consent from the participant in writing (if interviewed in person) or verbally (if interviewed via telephone) prior to the interview. Both interviewers were aged between 20 and 25 years, were female, and were trained in qualitative methods and interview techniques. HQ was a PhD researcher with a background in Sport and Exercise Psychology and BD was a medical student. With the participant’s consent, interviews were recorded using an Olympus Dictaphone. All interviewees were asked to speak freely and were assured that the interviews remained confidential.

The interviewers aimed to create a free-flowing discussion directed by the interviewee. An interview guide sought to explore HCPs’ perceptions of physical activity for children who have T1DM with open questions such as: *How do you think children with T1DM feel about taking part in physical activities?*; *How do you think having T1DM influences participation in physical activity? What could be done to help children with T1DM be more active?.*

Interviews were transcribed verbatim into Microsoft Word (Microsoft, Redmond, WA, USA), which facilitated early familiarisation with the data. Transcript analysis was an iterative process using thematic analysis [17]. This involved identifying codes, patterns (themes) and common threads across all transcripts. Codes were meaningful groups of data that captured the essence of the data. NVivo version 10 [18] was used to facilitate the organisation of codes and themes, and has been used previously in similar research [19].

Codes were derived primarily from the data (inductive) but could also be theory-derived (deductive) [17]. Codes arose through a deductive approach when the theoretical understanding found in the literature review allowed the researcher to be sensitive to certain topics that may arise in the data [16]. Examples of a priori codes were ‘*occurrence of hypoglycaemia*’, ‘*children’s concerns about hypoglycaemia*’ and ‘*parental fear of hypoglycaemia*’, as previous research has suggested that hypoglycaemia could be a potential barrier to physical activity. Inductive codes emerged from data and thus not anticipated in advance of data analysis. Data analysis began with an inductive approach. Deductive codes relating to specific areas of interest were then looked for in the data, but analysis was iterative rather than a rigid linear process.

Several approaches were used throughout the study to ensure methodological trustworthiness [20]. The researchers showed sensitivity, commitment and rigour (to theory, participants and data), transparency (e.g. being explicit with research decisions) and sought findings that would have practical implications. This was in addition to utilising a rigorous approach to establish the consistency and replicability of the themes [21]. A codebook was developed which included a brief background to the study, a label for each theme, a theme and sub-theme description and example extracts that did and did not illustrate each theme [21]. Quotes belonging to each theme were given to a second coder to code blind using the codebook. Boyatzis recommends that percentage agreement between two coders above 70 % demonstrates acceptable reliability [21]. The agreement was 89.5 %, indicating that the themes were consistent and reliable to a recommended standard [21].

Through reflective practice, the researcher was careful to acknowledge personal biases, values and judgements explicitly in a diary prior to and during the research process [17]. During data collection, the researcher made notes about the interview, including insights about the interview, participant and emerging points of interest. In the early stages of data analysis, the researcher noted impressions, ideas and initial interpretations of the data. This aided the generation of themes and served as a means for documenting the rationale for any changes or reassignment of codes and themes.

## Results

The purpose of this study was to explore HCPs’ perceptions of what influences physical activity for children with T1DM in an effort to inform those working with children who have T1DM. Factors believed to influence participation are presented as five major themes and eleven corresponding sub-themes. Verbatim quotes are provided to demonstrate themes, labelled with the participant’s professional role.

### Theme 1 Social support is a positive influence on children’s participation in physical activity

Social support was the most commonly identified influence on children’s physical activity, with parents and peers being perceived as important sources of practical and emotional support.

#### Parental responsibility and support is believed to be the key to children’s participation in physical activity

The majority of HCPs perceived parents as a powerful source of support for their child’s physical activity. Parental support encompassed parents being encouraging and demonstrating a positive attitude toward physical activity, for example:

“I think parental responsibility, so communicating with their children to say look, [physical activity] is important for you, for your development, to continue being social with your friends, obviously good for your health. So I think that parental role is extremely important, that supportive structure around them” (P09, Dietician)

Parental support was believed to be emotional (e.g., encouragement) and/or logistical (e.g., providing transport, “*they would need their parents to take them to the activity, Mum to drive them*” (P02, Consultant)). Some HCPs believed that it helped if parents were active and others believed an active parental role model was not necessary, for example, “*I don’t think necessarily that parents have to be really sporty to get the children to be sporty, but they encourage them to be sporty and take them to their hobbies and support them*” (P01, Dietician). Overall, parents were perceived as the key to children being provided the opportunity to be active.

“I suppose parents’ lifestyle influences…whether they have those opportunities to be active or whether their parents want to get on with other things and leave them to watch TV or play on the PlayStation” (P11, Dietician).

The parents believed to be less engaged and supportive were perceived as “*likely to be the most reluctant*” (P01, Dietician) to encourage their child to be physically active and less likely to prioritise the importance of physical activity:

“The main problem you're likely to face is not diabetes, it’s just, when are we [the family] going to fit this [physical activity] into our busy lives and is it really a priority?” (P03, Consultant).

#### Active friends are a positive influence on children’s physical activity

Active friends were deemed an influential support network for providing socialisation opportunities and modelling active behaviour. One HCP believed that participation in physical activity depended on, “*who they make friends with and whether they are into [physical activity], if they’ve got friends who play football they’ll go join them and play football after school*” (P11, Dietician).

### Theme 2 Characteristics of the child that enable participation

Specific characteristics of the child were thought to facilitate participation in physical activity. Whilst some biological factors were cited, such as age and gender, the most pertinent characteristic referred to the child’s motivation.

#### Individual motivation to be physically active is the main influence on children’s level of physical activity

Around half of the HCPs identified the child’s motivation to be physically active as the main influence on children’s participation in physical activity. Some HCPs believed that children are driven by “*what they’re interested in and what they feel they’re good at*” (P08, Dietician), implying that the motivation is intrinsic and participation in physical activity is likely to be for enjoyment and satisfaction. Others conceded that it is difficult to know what motivates some children: “*We don’t really know what it is that drives some people into it [physical activity] and others into couch potatoes*” (P11, Dietician).

Children involved in structured activity or organised sports were described as being the most motivated and committed, e.g., “*Taking part in competitive sport requires discipline anyway so you find that the patient and the family tend to be quite motivated and disciplined and that reflects on their diabetes control*” (P02, Consultant). Being active prior to T1DM diagnosis was perceived to coincide with the perceived ability of children to overcome barriers to physical activity, e.g., “*The ones that have always been active carry on and find a way to do that with the diabetes*” (P04, Nurse). The same nurse went on to say that these active children, “*know what they get out of the exercise already*” (P04, Nurse), which implies that experiencing some reward from previous participation can motivate children to be active after diagnosis.

### Theme 3 Formal organisations have the potential to support physical activity

The child’s school and healthcare team were identified as having the potential to influence children’s participation in physical activity.

#### Schools are believed to have a “*wonderful opportunity*” (P10, Consultant) to promote physical activity

School teachers were believed to have an important role in the facilitation of physical activity for children with T1DM. As a mandatory part of the school curriculum, Physical Education (PE) was believed to be an accessible opportunity for all children to be active “*whether they like it or not*” (P09, Dietician). For teachers supervising children with T1DM, “*the priority is safety*” (P09, Dietician) and HCPs perceived that it is the role of the diabetes team to ensure that schools are adequately informed and prepared to supervise and support pupils with T1DM via training and school visits.

“That has occasionally been an issue, where teachers haven’t understood or are frightened about what might happen and children are prevented from participating…well often the diabetes nurses can be quite helpful in those situations, going out to the school and talking to teachers, finding out their concerns and addressing those issues” (P03, Consultant).

#### Healthcare professionals’ role to educate and advise around physical activity

The majority of HCPs believed that it was their role to educate and support children with advice and guidance around physical activity; “*to give them the skills to be able to manage their diabetes to the best of their ability and perform that activity*” (P07, Dietician). They believed they should reassure parents that physical activity is safe when diabetes management plans are in place e.g., “*it’s important that we have a role …we reassure them that anything is possible as long as they’re willing to commit to what we say*” (P09, Dietician). And believed they were in a position to normalise the experience of hypoglycaemic episodes:

“We do tell the parents that having a couple of hypos a week is actually a sign of good control, as long as the child can recognise hypo symptoms…so it is normal as long as they’re just checking blood sugars and know how to treat them” (P01, Dietician).

Healthcare professionals described their tendency to discuss physical activity with specific children; overweight children e.g., “*if it’s a child who’s got a weight problem as well then we might address it*” (P02, Consultant) and children who were regularly active prior to diagnosis e.g., “*We talk about exercise if they’re sporty*” (P01, Dietician). Furthermore, some HCPs identified themselves or specific colleagues as being more inclined or suitable to give advice around physical activity. One HCP described their centre as being proactive in offering exercise advice to children; “*I think compared to other centres we are probably quite proactive in advising on exercise in diabetes*” (P04, Dietician) and a colleague in the same centre explained, “*I think it’s driven more by our personal interests as much as anything else*” (P03, Consultant).

#### Professional expertise supports the child’s existing lifestyle rather than promoting increased physical activity

The HCPs perceived that they were better placed to support the management of existing structured activities rather than promoting an increase in day-to-day physical activity. The management strategies described were individualised management plans, activity diaries and ongoing clinic discussions:

“With most patients you can find a pattern to say look the child tends to go a bit hypo maybe two hours after the activity, so we need to make sure that we give them a good carbohydrate meal, we cut down the insulin or work out a strategy that works” (P02, Consultant).

A minority of HCPs did promote lifestyle physical activity, describing how they encourage day-to-day activities such as walking to school and playing outside. One dietician perceived it easier to discuss physical activity with children who already had an interest being active, because then the HCP’s role was “*to support [the child’s] chosen lifestyle, but it’s very different to actively promoting physical activity in a group of people that you know will have problems*” (P11, Dietician). Another dietician acknowledged; “*people forget about anything that may just be sort of everyday activities…, walking to school say, and we concentrate a lot more on what we call ‘exercise’*” (P07, Dietician).

### Theme 4 Type 1 Diabetes presents specific challenges to physical activity

There was consensus among HCPs that T1DM “*shouldn’t really interfere*” (P01, Dietician) with day-to-day physical activities, but that structured, prolonged exercise and competitive sport participation need a diabetes management plan in place to ensure that participation is safe and performance is optimal. The current level of activity in children with T1DM was believed to be similar to their peers without diabetes. Nevertheless, HCPs recognised that diabetes presents unique challenges to children with T1DM engaging in active lifestyles.

#### Problems maintaining stable blood glucose control

Blood glucose control was perceived as a challenge for children with T1DM and their parents due to the extra demands of monitoring and managing fluctuating blood glucose levels around times of physical activity. The majority of HCPs perceived one of the main challenges to be the frequent testing of blood glucose level, which they sympathised as being “*difficult*” (P07, Dietician), “*boring*” (P04, Dietician), “*interfering*” (P01, Dietician) and “*a lot of effort*” (P01, Dietician). One HCP acknowledged that, “*I think our expectations of testing so frequently during physical activity are very difficult for people to keep up*” (P07, Dietician). Swimming and spontaneous activities were specific situations perceived to be problematic for maintaining a stable blood glucose level. Spontaneous activities are sporadic and typically unplanned, making it difficult for families or HCPs to pre-empt changes in blood glucose level:

“You’ve got a child…who suddenly decides to go out and bounce on the trampoline for half an hour and then their blood sugars go low and then it’s the parents that worry about sudden unpredicted exercise because that can make their sugars drop quickly” (P03, Consultant).

#### Parental concern regarding hypoglycaemia limits children’s physical activity

Every HCP interviewed accepted the negative impact that hypoglycaemia had on participation in physical activity for children with T1DM and agreed that the main challenge was the worry of hypoglycaemia, rather than its actual occurrence. Parental concern and worry about hypoglycaemia was the most commonly cited barrier to physical activity. One Consultant referred to parental worry as a normal response and “*part and parcel*” of being a parent of a child with T1DM, rather than a “*pathological worrying state*” (P02, Consultant). However, the same Consultant acknowledged; “*that [parental concerns about hypoglycaemia] will definitely limit the child’s participation*” (P02, Consultant). Nocturnal hypoglycaemia was identified as a specific cause of worry for parents:

“When they do become a bit sporty, they do struggle, especially with getting hypos, their blood sugars drop at some point in the evening after the activity and parents worry a lot about hypos in the evening or at night and that can be something that deters them from doing activities” (P02, Consultant).

A small number of HCPs perceived the child’s concerns about hypoglycaemia to be a potential barrier to physical activity; “*maybe it is that some of them are less confident because of the fear of hypos*” (P01, Dietician), but the general consensus was that “*it’s the parents that worry a lot more than the children*” (P08, Dietician). Some HCPs offered hypotheses for the cause of parental concerns about hypoglycaemia; parental worries due to a historical emphasis on the risk of exercise-induced hypoglycaemia, negative past experiences of hypoglycaemia, being newly diagnosed or parents worrying instead of the child; “*the younger kids, they probably just don’t have the awareness to worry about it [hypoglycaemia], so their parents worry on their behalf*“(P02, Consultant). No consistent reason was offered, but there was some agreement that most concerns are likely to follow an episode of hypoglycaemia, with the consequences for children being embarrassment or losing confidence and for parents having the lasting memory of the episode; “*[parents] have got the real scary situation in their head*” (P04, Specialist Nurse) and “*haunted them a long time later*” (P05, Dietician).

Four HCPs indicated that parents might avoid hypoglycaemia during or after physical activity by keeping blood glucose higher than is recommended, termed ‘maladaptive hypoglycaemia avoidance behaviour’. Amongst these HCPs, there was consensus that it was the role of the HCP to promote adequate levels of blood glucose rather than high levels:

“Often parents like to have their children have relatively high blood glucose levels and we try to listen to those concerns and empathise with them, but at the same time, we try to have them, rather than highs, we try to promote adequate levels” (P09, Dietician).

#### Concerns about diabetes being used as an excuse not to be active

Five HCPs believed that the extra effort required when a child with T1DM participates in physical activity could be used as an excuse not to participate, particularly by children who do not have a keen interest in being active; “*Sometimes the diabetes can be used as a nice convenient excuse but you usually find out that these were children who never did anything beforehand*” (P03, Consultant).

### Theme 5 Perceived barriers to healthcare professionals fulfilling their role to promote physical activity

The majority of HCPs could readily identify barriers to the successful promotion of physical activity in children with T1DM.

#### Healthcare professionals perceive difficulty implementing physical activity guidelines

Healthcare professionals confided that physical activity was not always a priority for discussion during clinic appointments, where time was prioritised to other aspects of diabetes care. The reasons suggested for not prioritising physical activity included; “*there’s quite a bit to it*” (P10, Consultant), and “*you only get a small amount of time*” (P09, Dietician). Healthcare professionals found it difficult to translate physical activity information into a comprehensible format e.g., “*difficult trying to translate that information into a digestible form for children”* (P11, Dietician). Two HCPs suggested that the limited time available during routine clinic appointments meant that details around physical activity promotion and management were often omitted. Also, it was acknowledged that the effective implementation of guidelines was dependent on there being commitment from the child and family:

“If people are really going to manage their diabetes well during exercise that takes a lot of commitment in terms of what we may ask people to do. We might ask them to take blood glucose before and then every 20-30 minutes during activity and every hour afterwards” (P07, Dietician).

#### Healthcare professionals acknowledge the need for further training and resources

Healthcare professionals believed that standardised guidelines for physical activity participation would be beneficial to educate children with T1DM and their families, however conceded that “*there's not an off-the-peg solution to anything*” (P11, Dietician). Instead, because advice needs to be tailored to the individual child, its effectiveness depends on the ability of parents to understand how blood glucose levels respond to physical activity; “*It can be quite individual for the patient and that can be quite overwhelming*” (P07, Dietician).

There was general agreement that resources or referrals were available for children participating in structured or high level exercise and sport, however a gap was perceived in the availability of resources to promote and manage everyday lifestyle physical activities.

“It would be useful to get better resources…a nice hand-out that we could actually give out UK-wide would help families get the right support and education they need and make sure all centres are giving the same advice” (P05, Dietician).

Another HCP suggested that a curriculum or resource aimed at school teachers would be useful, especially PE teachers, who are “*ideally placed to understand*” (P10, Consultant) exercise:

“It might be useful to have a bit of a curriculum that is clear, directed at teachers for example, with a bit more for those that do sporting activities…they will be the ones to have a child go hypo if that’s not properly planned or monitored” (P10, Consultant).

Two HCPs described initiatives that had been developed in their respective centres to address this need for resources and facilitate the discussion and management of physical activity; i) an algorithm (not yet evaluated) giving instructions to children depending on their blood glucose level prior to physical activity and ii) an education programme for adolescents making the transition from paediatric to adult care [22].

A small number of HCPs lacked confidence in their ability to implement physical activity guidelines and questioned the effectiveness of the guidelines they were implementing: “*I’m not quite sure how effective that education is*” (P10, Consultant). Some suggested that further training might facilitate the promotion and management of physical activity in the clinic setting; “*I don’t always feel I know all I need to know about it…and so I think educating health professionals is a starting point*” (P07, Dietician), and; “*I don’t feel adequately informed, probably because I haven’t studied [physical] activity*” (P11, Dietician). One dietician described a self-initiated solution to this lack of mainstream physical activity training was to attend physical activity conferences:

“We don’t get enough training as a dietician you don’t get any training in Type 1 Diabetes particularly until you start doing it let alone on sports and exercise. So the way I’ve been trained is because I’ve gone to conferences on specific days and I’ve gone out my way to do that; it’s not an essential part of the training” (P05, Dietician).

## Discussion

Interviews indicated that HCPs involved in the care of children with T1DM perceived they had an important role in providing physical activity advice and support to children with T1DM and their parents. There was agreement that diabetes should not be a barrier to children participating in any physical activity, exercise and sport. Yet challenges were identified that were perceived to make regular participation problematic for some children with T1DM. Themes demonstrated perceived facilitators and barriers that are shared with the general population of young people, including the positive influence of social support, the child’s motivation to be active and the potential for formal organisations such as school to promote and support active lifestyles. Themes alluded to the specific role of the diabetes team and teachers to support physical activity among children with T1DM, the challenges faced by children with T1DM and their parents, and the perceived barriers to HCPs fulfilling their role of promoting physical activity.

The findings demonstrate how HCPs perceived parental support to be an important influence on children’s physical activity participation. Of particular importance was parents’ emotional and logistical support. Parental factors, family life and the home environment have been shown to influence a child’s physical activity behaviour [23] and specifically the positive influence of parents’ emotional [24] and logistical [25] support. Parents are often responsible for the day-to-day management of blood glucose control in children with T1DM, which suggests that they may have a unique influence on their child’s physical activity behaviour. Previous research [13, 26] has identified the important role of parents in the physical activity participation of children with T1DM. We have shown that HCPs perceive parents to be one of the main sources of social support for children with T1DM engaging in regular physical activity. This suggests that parents should be targeted as influential agents in any attempt to promote physical activity in children with T1DM. Similarly, HCPs identified children’s friends as important for promoting participation in physical activity, particularly their active friends. Previous research suggested that peer support could be an important component of physical activity interventions for children with T1DM [13]. Bandura’s SCT [12] proposed that individuals learn behaviours by observing and imitating others through vicarious experience and the current findings imply that active friends could serve as important role models to children and could be targeted in attempts to promote physical activity in children with T1DM.

We found that children’s individual preference for physical activity was perceived to be an important influence on their uptake and maintenance of physical activity. In particular, HCPs observed how children’s enjoyment of physical activity was related to their history of physical activity participation and accomplishment. This suggests that children might be intrinsically motivated to engage in physical activity. Previous research identified intrinsic motivation [27] and enjoyment [28] as positively associated with children’s participation in physical activity. Mastery experiences, which involve some previous successful accomplishment, are proposed to be an antecedent of self-efficacy [12]. Self-efficacy could be a powerful drive influencing children’s motivation and has been identified as a psychological determinant of children’s physical activity participation [29]. These findings suggest that attempts should be made to uncover what motivates children with T1DM to be physically active and to foster children’s self-efficacy for physical activity through fun and enjoyable ways to keep active.

Schools were perceived to have a valuable opportunity to enable and promote physical activity among children with T1DM, but this was dependent on teachers being trained and prepared to supervise children’s physical activity. Previous research highlighted that teachers, youth with T1DM, their parents and diabetes professionals valued the importance of teachers’ T1DM knowledge and training for encouraging children with T1DM to be physically active in school [6]. Parents, in particular, have expressed the importance of competent and supportive school teachers [10]. The HCPs believed that it was the role of the diabetes team to ensure that training and safety precautions are in place at school to facilitate physical activity and sport engagement in children with T1DM. However, this would not only rely on healthcare teams having the capacity to deliver intensive training to school teachers but whether they feel confident in doing so. It is arguable as to whether there is a need for specific training on the management of long-term conditions such as diabetes as part of teacher training. In addition, whilst PE in schools was valued by HCPs for its widespread accessibility, it must be acknowledged that classes may occur infrequently and children may be relatively inactive during the class. This raises the issue as to whether schools can do more to promote physical activity throughout the course of the day [30]. The notion of school teachers promoting physical activity in children with T1DM is consistent with MacMillan et al. (2014), who provide guidance on what teachers can do to support children with T1DM being active in school [6].

Healthcare professionals perceived themselves and their colleagues involved in the care of children with T1DM to be an important source of support for children with T1DM engaging in physical activity. They described their role in *facilitating* the management of children’s existing activities and sport participation rather than *actively promoting* physical activity in children’s daily lives (e.g., walking to school). Our findings suggest that HCPs recognised that their role was to reassure children and parents about physical activity, but admitted that their influence might be limited to those who were ‘sporty’ or had an existing interest in physical activity. Paradoxically, the children who would benefit the most from increased physical activity were perceived as the most difficult for HCPs to engage in conversation around physical activity. This goes some way to explain previous research findings that health professionals do not perceive themselves to be influential in the physical activity participation of children with T1DM [13]. Health professionals and policy makers may need to think beyond traditional sports and activities and consider the promotion of active lifestyles among children, especially those who may not have been active prior to their diagnosis.

The HCPs appreciated that the demands of managing blood glucose levels could be a deterrent to physical activity, especially in those children who lacked interest in or motivation for physical activity. Children typically engage in spontaneous, intermittent bouts of activity and this was perceived to be challenging because the fluctuation in blood glucose level cannot be anticipated or offset with pre-planned insulin or dietary adjustments. Parental fear of hypoglycaemia was a commonly cited barrier to physical activity perceived by HCPs, but was not considered a maladaptive worrying state. Given the potential danger of low blood glucose levels, some degree of fear around hypoglycaemia is considered appropriate and adaptive [31]. Health professionals involved in the care of children with T1DM should seek to uncover concerns and give children and parents the skills and confidence to manage hypoglycaemia during and after physical activity.

The HCPs in this study believed they had a role in the promotion and management of physical activity, but identified aspects of their work conditions that made it difficult to fulfil this role. Some HCPs lacked confidence in either their own physical activity knowledge or the information available to them, which suggests they might be inadequately trained to deliver the guidance. They also perceived difficulty in implementing physical activity guidelines, and identified barriers to doing so, such as time constraints, translating the advice into a digestible format and feeling inadequately equipped to deliver the advice. The barriers identified by the HCPs were consistent with those identified by medical professionals in other health domains, including; time constraints [32], their own interests and health behaviours [33], lack of standard protocols and lack of financial incentive [33, 34]. This consistency suggests that our findings may have implications for the promotion of physical activity across the population. Healthcare systems are natural settings for the promotion of physical activity as they often involve repeated contact between HCPs and patients [35]. We do not understand whether advice to engage in physical activity given by HCPs is effective in changing children’s behaviour. It would not be unreasonable to infer from our findings that HCPs might benefit from training opportunities to foster competence in the implementation of guidelines, promotion and management of physical activity. Diabetes teams might benefit from having a staff member who is specifically trained in physical activity advice and guidance and who has the confidence to champion physical activity promotion within the clinic. The effectiveness of this approach could be explored in future research.

The high consistency of themes supports the credibility of the findings and ongoing reflective practice enhances the methodological rigour. However, the findings should be considered in light of the following methodological issues. The HCPs were self-selected and therefore the study may have reached those with a personal interest in physical activity. Self-selection, together with snowball techniques meant that the HCPs in this study may have held different perceptions to those HCPs who were not interested or able to talk about physical activity for children with T1DM. However, a range of opinions about physical activity seemed to be captured. Also, the combination of telephone and face-to-face interviews meant there was methodological disparity which may have elicited different responses. However, telephone interviews allowed for a more diversity in participant type and geographical location.

## Conclusions

The findings raise awareness of the difficulties faced by children with T1DM in relation to physical activity, and highlight the potential for clinical and non-clinical supportive systems to be sensitive to these challenges and facilitate children’s regular participation.

## Abbreviations

T1DM: Type 1 diabetes mellitus
HCP(s): Healthcare professional(s)
SCT: Social cognitive theory
PE: Physical education

## Footnotes

1 The phrase “children and young people” has been referred to as “children” throughout.
